# Prolonged elevation of viral loads in HIV-1-infected children in a region of intense malaria transmission in Northern Uganda: a prospective cohort study

**Published:** 2010-11-09

**Authors:** Herbert Samuel Kiyingi, Thomas Gordon Egwang, Maria Nannyonga

**Affiliations:** 1 Homecare Department, St. Raphael of St. Francis Nsambya Hospital/Elizabeth Glaser Pediatric AIDS Foundation Collaboration; 2 Centers for Disease Control and Prevention, Uganda

**Keywords:** HIV infection, Plasmodium falciparum, Malaria, children living with AIDS, viral loads

## Abstract

**Introduction:**

Malaria and HIV-1 infection cause significant morbidity and mortality in children in sub-Saharan Africa. Recurrent malaria infection increases HIV-1 viral load in adults and increases the rate of progression of HIV-1 infection to AIDS. The effect of malaria on viral loads in children living with AIDS (CLWA) is not clearly known.

**Methods:**

One hundred thirty five afebrile HIV-1 positive children having negative blood slides for malaria were recruited at Apac Hospital and followed up for one year. They were monitored for development of *Plasmodium falciparum* malaria, which was treated with chloroquine (CQ) + sulfadoxine-pyrimethamine (SP) and the children followed up for 28 days. HIV-1 viral loads were measured over three time-points: at enrolment (no malaria), during an episode of malaria, and at a visit about 8 weeks (range 6-19 weeks) after the malaria visit when the child had neither parasites nor any intervening malaria episodes (post-malaria). Primary analyses were restricted to children who on follow up had HIV-1 viral loads measured at the three relevant time-points.

**Results:**

Malaria increased HIV-1 viral load significantly in CLWA. Low parasitemia (200-4000/Cl) transiently increased viral load by 0.42 log (95% CI 0.29-0.78, p = 0.0002), higher than that reported in adults. These patients’ viral loads returned to levels similar to those at baseline after treatment. In 13 patients with high parasitemia (>4000/Cl), the mean increase in viral load was 0.53 log (95% CI 0.14 to 0.51), p<0.0001, remaining significantly higher than at baseline after treatment i.e. mean difference (signed-rank test) in viral load “before” and “after” malaria was significant.

**Conclusion:**

*Plasmodium falciparum* malaria is associated with increasing HIV-1 viral loads in children, with some viral loads remaining significantly elevated several weeks after antimalarial treatment. Prolonged post-treatment elevation has important implications for the clinical course in pre-ART HIV-1 positive children and the potential for transmission in sexually active adults.

## Introduction

There are currently approximately 140,000 Ugandan children living with HIV-1/AIDS (CLWA) [1], of whom less than 15% have access to antiretroviral drug treatment (ART) [1]. Despite concerted international and national efforts, universal access to ART has still remained elusive due to logistic and other implementation problems. At least 50% of HIV-1 positive children without access to ART die by the age of 2 years in Uganda [2]. HIV and malaria have similar global distributions, with sub-Saharan Africa being the hardest hit. 80% of malaria deaths globally occur in children. Malaria caused by *Plasmodium falciparum* remains a significant public health problem responsible for about 30% of all hospital admissions and 110,000 infant deaths annually in Uganda [1]. Minor effects of one infection on the disease course or outcome for the other would significantly impact public health because of the sheer number of people at risk for coinfection. The effect of malaria on HIV infection is not as well established in children; who suffer the largest burden of malaria in sub-Saharan Africa. There is evidence that HIV-1 RNA concentration is higher in adult patients with *P. falciparum* malaria than in controls and that this viral burden can be partly reduced with antimalarial therapy [3,4]. Since higher viral loads are associated with disease progression, it is important to investigate whether or not malaria clinical episodes in CLWA increase HIV-1 viral loads and thereby predispose to progression to full blown AIDS. Apac district is located in Northern Uganda. Malaria transmission in Apac is throughout the year with two transmission peaks during the rainy seasons [12].

We undertook a comparison of HIV-1 RNA concentrations at baseline (before malaria episodes), during malaria, and post-malaria (several weeks after curative antimalarial treatment or discharge from the hospital), and determined the values of malaria-mediated increases in HIV-1 RNA.

Our goal therefore was to measure the effect of *P. falciparum* malaria on HIV-1 viral loads in Ugandan children living with HIV/AIDS but who are not yet on anti-retroviral Therapy. The study was done over a period of 18 months.

The study site was Apac Hospital located in Apac District of Northern Uganda. Sixty percent of the district is swampy, while the rest of the terrain is savanna vegetation which provides ideal habitats for mosquito breeding. This site has one of the highest malaria transmission intensity in the world, with an Entomological Inoculation Rate of 1586 [9] i.e.: Six bites per person per night by a malaria transmitting mosquito; making malaria endemic in this area. This government hospital also provides psycho-social support and palliative treatment and care to more than 20,000 people living with HIV-1/AIDS including 300 children under 12 years old. All the HIV positive children receive cotrimoxazole prophylaxis against *Pneumocystis jiroveci* infections as per the existing National HIV care guidelines [8]. We recruited subjects and did the clinical aspect of the study from this site. Since cotrimoxazole has antimalarial activity, this confounding variable was taken into consideration during data analysis.

Our hypothesis was that *P. falciparum* infections increase HIV-1 viral loads CLWA; which in turn increases the rate of disease progression to AIDS in ART naïve children.

## Methods

The study population was ART naïve HIV-1+ children aged 18months to 12 years, with CD4 counts > 15% who were attending Apac hospital. These children all received cotrimoxazole prophylaxis for *Pneumocystis jeroveci* pneumonia and were not yet on ART. Ethical approval was obtained from the IRBs of Medical Biotechnology Laboratories, St. Francis Nsambya Hospital, Apac Hospital and the Uganda National Council for Science and Technology, and informed consent was elicited from the parents and guardians of all study subjects. In order to remove confounding factors known to affect viral load measurements in children, we excluded from the study children who had clinical features defining AIDS, a recent history of tuberculosis or demonstrable worm infestation, bacteremia, or suspected bacterial pneumonia or any other febrile illness. Children whose parents refused to give consent, and those who were not able to remain in the study for at least 9 months from enrolment were also excluded.

One hundred thirty five afebrile, malaria-free, and HIV-1 positive (by at least two rapid diagnostic assays) children who were 1.5 -12 years old were enrolled before the peak malaria transmission period (baseline). We used Abbott Determine and Unigold as the rapid HIV test kits. The children were followed up through the malaria season using a prospective cohort design to assess the effect of malaria on plasma HIV-1 RNA. Children who presented to the Outpatient Department with mild malaria or were hospitalized with severe and complicated malaria during follow-up received a standard dose of chloroquine (CQ) (Avloclor, ZENECA, 10 mg/kg on days 0 and 1, 5 mg/kg on day 2) plus a single dose of 1.25mg/kg pyrimethamine and 25 mg/kg sulfadoxine (Fansidar, Roche) on day 0; or quinine (10mg/kg), respectively, according to national guidelines. All doses were directly observed and if a patient vomited within thirty minutes of dosing, the medication was re-administered. Paracetamol was administered to all patients. The first dose of CQ+Fansidar was given at the clinic; the remaining 2 doses of CQ were given at home by parents/guardians.

Monitoring: Mothers or guardians were instructed to follow a schedule of hospital visits that included a baseline visit (Day 0), routine visits every 8 weeks, interim visits when the children were ill, and visits 3, 7, 14, and 28 days after anti-malarial treatment. After 28 days, the children returned for their regularly scheduled routine visits until the end of follow up. Mothers were given a transport refund on each visit to the clinic.

HIV-1 viral RNA was measured over three time-points: at enrolment (baseline, no malaria), during an episode of malaria (malaria), and a visit about 8 weeks after the malaria visit and the child had no parasites nor had any intervening malaria episodes (post-malaria). Primary analyses were restricted to HIV-1 positive children who were afebrile and parasite-free at baseline, had follow up visits, had at least one episode of *P. falciparum* parasitemia during follow up, and had HIV-1 viral loads measured at the three relevant time-points. HIV-1+ children without malaria parasites at enrolment and who remained aparasitemic throughout the study served as a comparison group; they were followed up in parallel with HIV-1 RNA measurements at the 3 corresponding time points.

**Clinical evaluation**

At enrolment, baseline demographic data was collected. At each hospital visit, a physical examination was carried out and a blood sample collected for complete leucocyte counts, malaria microscopy, and HIV-1 RNA load measurement. A history of fever or a sublingual temperature of > 37.5 °C was investigated by clinical examination, blood film examination for malaria parasites, differential WBC counts, were done. Four case definitions of malaria were used: any parasitemia, parasite density 10-20/High Power Field (HPF), parasitemia + fever, and parasite density V 20/HPF + fever. CD4 counts were done as per the schedule of the clinic. Milestone-defining CD4 counts; i.e. counts less than 15% of the total lymphocyte count, were flagged. These thresholds, which signal when to start or change ART, were based on reference interval data for infants on ART from other Ugandan settings and data extrapolated from pediatric AIDS children in neighboring Kenya [13].

**Laboratory analyses**

Thin and thick blood films were made for each child at baseline, interim visits and post malaria treatment. The films were processed, Giemsastained, and examined for malaria parasites. A rapid microscopic examination of thick films was carried out at the hospital to confirm the clinical diagnosis of malaria (malaria parasite-positive slides) so that children with confirmed malaria were promptly treated with antimalarial drugs. The thin slides were examined under oil immersion at high magnification (100 X) to determine the parasite density. The number of asexual parasites was counted against 200 white blood cells (WBC) per high power field. The number of asexual blood stage parasites/µl was calculated as the mean number of parasites counted per high power field multiplied by 40 on the assumption of an average white cell count of 8,000 cells/ µl [14]. Total leucocyte counts were carried out using a Cell Dyn 1800 automatic hematology analyzer (Abbott Diagnostics, USA) . HIV-1 RNA was measured by PCR (Amplicor HIV-1 Monitor 1.5 assay, Roche Molecular Systems).

**Data analysis**

Data was double-entered and validated using EpiInfo (version 3.5.1). The analysis was carried out using STATA software, version 7.0 (College Station, TX, USA). The study was designed to identify a minimum 0.5-log increase in mean HIV-1-RNA concentration during a clinical episode of malaria, with two-tailed Y= 0.05 and Z = 0.80. HIV-1 RNA was log10 transformed before analysis. Baseline characteristics of the population and characteristics between groups were compared with Pearsons X2 tests for categorical variables. All comparisons were 2-tailed. A multivariable analysis was also conducted to determine the impact of other non-malaria comorbidities on the observed effect. Clinical and demographic variables were evaluated in univariate repeated measures analysis to determine associations with log-transformed HIV-1 viral load.

## Results

A total of 210 HIV-1 positive children seen at the pediatric HIV clinic were screened for malaria. 25 (12%) had a positive malaria blood smear at screening and were referred for treatment and excluded from the study. 135 fulfilled the inclusion criteria and were enrolled into the study.

**Figure 1:**
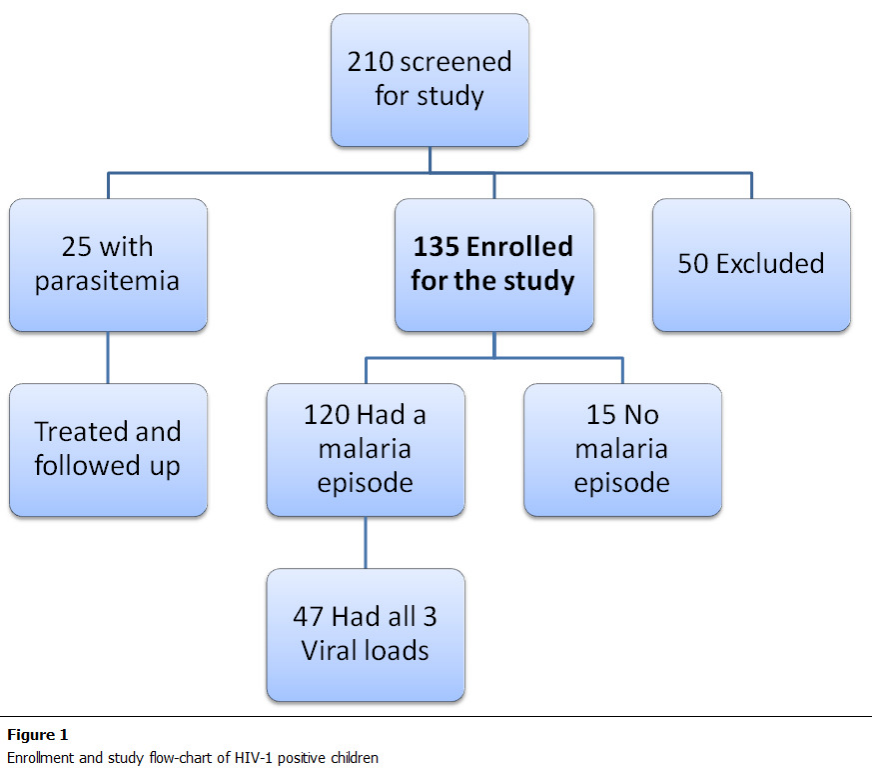
Enrollment and study flow-chart of HIV-1 positive children in the study

The primary reasons for exclusion were: presence of concomitant febrile illness i.e. a febrile child with two negative Blood Smears at least 6 hours apart, but has other foci of infection like pneumonia, flu, UTI, wound sepsis, GE or a neutrophilia. (16, 32%), not able to continue through the study period (13, 26%), AIDS defining illness (6, 11%), and malaria (15, 30%) (Figure 1).

Of the 135 HIV positive children recruited, 120 patients had at least one malaria episode during follow up and received antimalarial treatment. This was a high incidence rate of about 11 cases of smear positive malaria per child-month of follow up time. Of these, 47 had viral load measurements at all three key time points. 15 patients did not get malaria. The baseline characteristics of patients who completed the study and were included in the analysis are shown in table 1. Baseline characteristics of HIV-1 positive children were comparable, with 27 females (60%) and 20 males. HIV-1 patients less than 5 years old were 45% of the study population.

**Figure 2:**
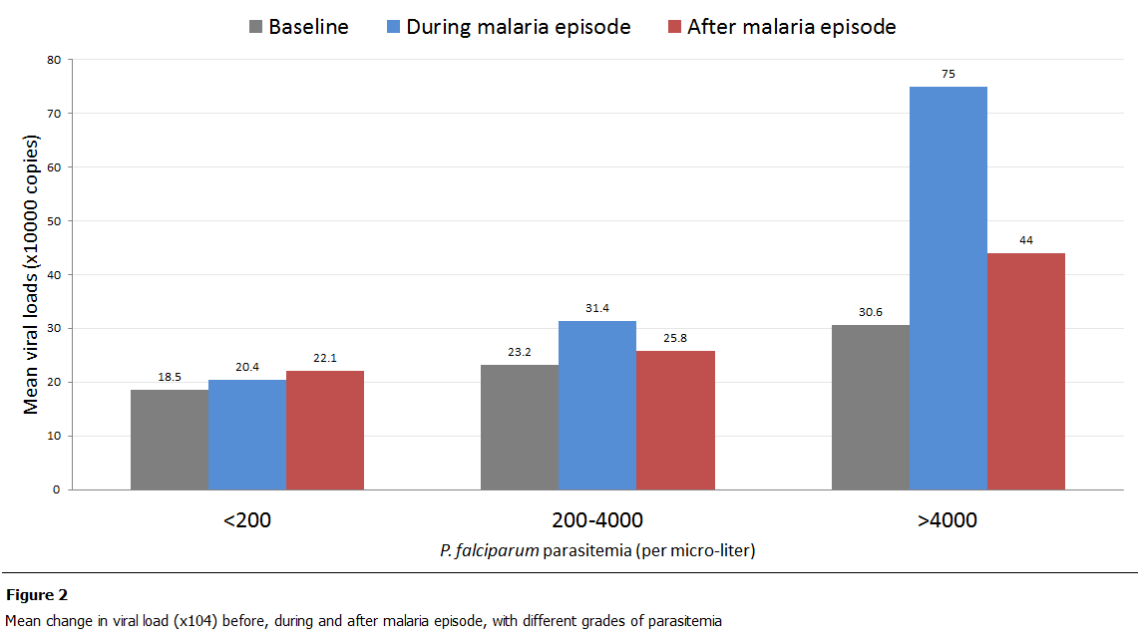
Mean change in viral load (x104) before, during and after malaria episode, with different grades of parasitemia

The median viral load of the patients used in the analysis was 55,000copies/ml (table 2). The average time to developing a malaria episode during follow up was 76 days. There is a general trend showing a transient increase in viral load during the malaria episodes throughout the study population (Figure 2).

When malaria was defined simply as parasitemia <=5/HPF, episodes were found to transiently increase viral load by 0.42 log (95% CI 0.29–0.78, p = 0.0002 within this stratum). About 8 weeks (range 7-16) after treatment for malaria, the patient’s viral loads returned to levels similar to those at baseline. The effect on HIV viral load increased with the severity of malaria. In 13 patients with fever and high parasitemia (10-20/HPF), the mean increase in viral load was 0.53 log (95%CI 0.14 to 0.51), p = <0.0001 as compared to those with low parasitemia. However, it was noted that in cases with a high parasitemia, the viral load still remains significantly higher than at baseline even after treatment i.e. mean difference (signed-rank test) in viral load “before” and “after” malaria was significant. Baseline viral load was a strong predictor of load during malaria.

We were not able to show data of the CD4+ counts of the children during the study because the counts were not done regularly by the hospital as we had anticipated. It was therefore difficult to have this data at comparable time lines.

## Discussion

The high malaria incidence in the study population may be attributed to the fact that recruitment was intensified during the two peaks of malaria transmission in Apac district. These findings suggest that *P. falciparum* malaria infection affect plasma HIV-1 levels in children as had been demonstrated earlier in adults [3,4,27]. While clinical malaria leads to at least short term HIV viral load increases in adults [4,27], children show a more marked increase. However, Children usually have higher baseline viral loads than adults. The effect of sub-clinical malaria remains unclear, since malaria diagnosis was confirmed by microscopy instead of PCR, and therefore, children with sub-clinical malaria were excluded from this study and as such missed. Also, negative slides after treatment did not rule out 202 sub-clinical malaria. However, lack of fully acquired antimalarial immunity in children may explain the difference in HIV/malaria interactions as compared to those seen in adults.

There are a few limitations to this study. First, all the CLWA were on cotrimoxazole prophylaxis for PCP. Cotrimoxazole, by inhibiting the dihydrofolate reductase (DHFR) of *P. falciparum*, has some antimalarial action [15-17]. Cotrimoxazole prophylaxis might therefore have reduced the incidence of malaria clinical episodes in CLWA and its impact on HIV-1 viral loads. However, cross-resistance between sulfadoxinepyrimethamine (SP), which also targets *P. falciparum* DHFR, and cotrimoxazole has been reported [18]. Although we do not have resistance data in Apac, we speculate that clinical episodes of malaria in CLWA on cotrimoxazole prophylaxis might be due to “break through” resistant parasites and that it is these parasites which might cause life-threatening alterations in HIV-1 RNA loads.

Second, the fact that the remaining chloroquine doses were given at home might have resulted in poor compliance and prolonged the “during malaria” period and as such prolonged the effect of malaria on the viral load. Third, infections with other pathogens such as tuberculosis, bacterial pneumonia, and helminthic infections might also have increased HIV-1 RNA concentrations. Although children with these and other infections were rigorously excluded the effect of subclinical conditions could not be completely removed and therefore the need for a multivariate analysis to determine the impact of these comorbidities.

Fourth, there is concern over the wide range of ages of the study population. There might be some expected variation in the progression of disease over the range 5-12 years. However, the sample size was too small to allow for any reasonable stratification by age. The lack of comparable CD4+ counts makes it difficult to conclude whether the baseline immunological state has a bearing on the severity of the malaria and the log increase in viral load. Our assessment was limited in size and duration of the study. Furthermore, in attempting to provide optimal patient care through conducting monthly surveillance and encouraging mothers to bring children in during febrile episodes, together with the fact that these children were all on cotrimoxazole prophylaxis, our ability to assess the effect of high-density malaria was diminished because parasitemia levels reached very high significant levels in only four cases. The lack of facilities to diagnose HIV-1 infection early in children below 18months meant that this age group could not be included in this study. Although malaria is not common occurrence in this age group, due to maternal antibodies, a large amount of the antibodies are transferred through breast milk [28,29] and yet some of these mothers will not breast-feed as a PMTCT measure [30]. It therefore would have been good to study the effects in a non-breastfeeding group as well.

The fact those in some children viral loads remain significantly elevated after anti-malarial treatment has important implications for the clinical course of HIV and its progression to AIDS in a setting where antiretroviral drugs are still not readily available. Since clinical malaria in children increases HIV-1 viral load as it does in adults [4,27], this study then provides proof of principle and lays ground to assess the value of intermittent presumptive treatment IPT of malaria in CLWA .It also emphasizes the importance of malaria control programs in the combined fight against HIV/AIDS in patients, especially children.

## Conclusion

The fact those in some children viral loads remain significantly elevated after anti-malarial treatment has important implications for the clinical course of HIV and its progression to AIDS in a setting where antiretroviral drugs are still not readily available. Since clinical malaria in children increases HIV-1 viral load as it does in adults [4,27], this study then provides proof of principle and lays ground to assess the value of intermittent presumptive treatment IPT of malaria in CLWA .It also emphasizes the importance of malaria control programs in the combined fight against HIV/AIDS in patients, especially children.

## Conflicts of interest

The authors declared they have no conflict of interest.

## Authors’ contributions

The primary author developed the concept of the study. The co-authors took part in the study design and assisted interpretation of the results. All clinical work and data collection for the study was done by the primary author. The writing of this paper was done by the Primary author assisted by the co-authors. The primary author of this paper did this study while working with St. Francis hospital Nsambya as a clinical research fellow under Elizabeth Glaser Pediatric Foundation collaboration. Although he currently works with the US Centers for Disease Control and Prevention (CDC), the CDC had no role in the study design, implementation nor data analysis.

## Figures and Tables

**Table 1:** Distribution of baseline demographics in 135 HIV-1 positive children participating in the study (Number, percentage)

**Sex**	**Male**	56 (41.1% )
	**Female**	79 (58.9%)
**Age (years) %**	1.5 to 5 years	60 (44.5%)
	5 to 12 years	75 (55.5%)
**WHO classification**		
**Sex**	**Male**	**56 (41.1% )**
	**1**	80 (59.3%)
	**2**	55 (40.7%)
**Median Hemoglobin (g/dl)**	10.5	(range: 9.8 – 13.8)

**Table 2:** Frequency of different grades of parasitemia observed in HIV-1 positive children participating in the study population

**Parasitemia (per <liter)**	**N**	**Median viral load baseline (A) x 104copies (95%CI)**	**Median Viral load after Malaria (B)x104copies (95% CI)**	**Log difference in median viral load log(B-A)**	P*
**Less than 200**	34	18.5 (14.3-20.1)	23.5 (17.9- 25.7)	4.70	
**200- 4000**	10	23.4 (19.1- 26.6)	26.8 (20.1- 28.7)	4.53	0.0005
**>4000**	3	32.9 (25.5- 44.4)	46.1 (36.7- 50.2)	5.12	4

N: Number of patients, *: Wilcoxon signed ranks test for Median difference
